# An anchor-based YOLO fruit detector developed on YOLOv5

**DOI:** 10.1371/journal.pone.0331012

**Published:** 2025-09-05

**Authors:** He Honggang, Olarewaju Mubashiru Lawal, Yao Tan, Kui Cheng

**Affiliations:** 1 Sanjiang Institute of Artificial Intelligence and Robotics, Yibin University, Sichuan, China; 2 School of Mechanical and Electrical Engineering, Yibin University, Sichuan, China; State University of Feira de Santana: Universidade Estadual de Feira de Santana, BRAZIL

## Abstract

Fruit detection using the YOLO framework has fostered fruit yield prediction, fruit harvesting automation, fruit quality control, fruit supply chain efficiency, smart fruit farming, labor cost reduction, and consumer convenience. Nevertheless, the factors that affect fruit detectors, such as occlusion, illumination, target dense status, etc., including performance attributes like low accuracy, low speed, and high computation costs, still remain a significant challenge. To solve these problems, a collection of fruit images, termed the CFruit image dataset, was constructed, and the YOLOcF fruit detector was designed. The YOLOcF detector, which is an improved anchor-based YOLOv5, was compared to YOLOv5n, YOLOv7t, YOLOv8n, YOLOv9, YOLOv10n, and YOLOv11n of YOLO variants. The study findings indicate that the computation costs in terms of *params* and *GFLOPs* of YOLOcF are lower than those of other YOLO variants, except for YOLOv10n and YOLOv11n. The *mAP* of YOLOcF is 0.8%, 1.1%, 1.3%, 0.7%, and 0.8% more accurate than YOLOv5n, YOLOv7t, YOLOv8n, YOLOv10n, and YOLOv11n, respectively, but 1.4% less than YOLOv9t. The detection speed of YOLOcF, measured at 323 *fps*, exceeds that of other YOLO variants. YOLOcF is very robust and reliable compared to other YOLO variants for having the highest *R*^*2*^ of 0.422 value from count analysis. Thus, YOLOcF fruit detector is lightweight for easy mobile device deployment, faster for training, and robust for generalization.

## Introduction

It is no news that the application of computer vision with deep learning to fruit detection has gained a lot of attention and acceptance. This is in response to fostering fruit yield prediction, fruit harvesting automation, fruit quality control, fruit supply chain efficiency, smart fruit farming, labor cost reduction, and consumer convenience. You Only Look Once (YOLO), as one of the detector algorithms generally harnessed, has shown to be highly prospective in this regard, bringing about its improvement from version to version over the years for having various YOLO-mainstream variants.

The YOLOv3 (Redmon and Farhadi, 2018) [1], which used DarkNet53 backbone with Leaky ReLU (Mass *et al*., 2013) [2] activation, feature pyramid network (FPN) (Lin *et al*., 2017) [3] as Neck, and binary cross-entropy loss, was improved for kiwifruit detection in orchards by Fu *et al*. (2021) [4], muskmelon detection by Zheng *et al*. (2019a) [5], and tomato detection by Liu *et al*. (2020) [6]. The modification of YOLOv3 for generalized object detection led to the introduction of YOLOv4 (Bochkovskiy *et al*., 2020) [7]. YOLOv4 includes the CSPDarknet53 Backbone with Mish (Diganta Misra, 2019) [8] activation, spatial pyramid pooling (SPP) (He *et al*., 2015) [9], Path Aggregation with Feature Pyramid Network (PAFPN) (Liu *et al*., 2018a) [10] as Neck, and YOLOv3’s Head. The improved YOLOv4-tiny for fruit and vegetable detection was published by Latha *et al*. (2022) [11]; likewise, Parico *et al*. (2021) [12] reported for real-time pear fruit detection and counting, Tang *et al*. (2023a) [13] detected each camellia oleifera fruit target in an orchard, and Mei-Ling and Yang (2023) [14] suggested GCS-YOLOv4-tiny to detect different growth stages of fruits.

The pursuit of a faster detection speed resulted in the development of YOLOv5 by Jocher *et al*. (2022) [15]. YOLOv5 adopted the CSPDarknet53 Backbone that contains C3 modules with SiLU (Stefan *et al*., 2017) [16] activation, Spatial Pyramid Pooling Fast (SPPF), and improved PAFPN as Neck. As part of YOLOv5 improvement for fruit detection, Zhang *et al*. (2022) [17] added ghost network by Han *et al*. (2020) [18] to detect a dragon fruit in the natural environment, Gai *et al*. (2021) [19] reported YOLOv5s-cherry for cherry detection, Xu *et al*. (2023) [20] lunched YOLO-Jujube to detect jujube fruit automatically for ripeness inspection, Lawal (2023a) [21] developed a lightweight YOLOStrawberry for strawberry detection, Qiao *et al*. (2021) [22] incorporated ShuffleNetv2 by Ma *et al*. (2018) [23] for a counting method of red jujube, and Lawal *et al*. (2023b) [24] applied feature concatenation with coordinate attention mechanism (CAM) introduced by Hou *et al*. (2021) [25] to detect fruit. With special attention to the complex environment of greenhouses, YOLOv4 and YOLOv5 were improved by Lawal (2024) [26] to detect cucurbit fruit in real-time. Meanwhile, YOLOv6 (Li *et al*., 2022) [27] introduced EfficientRep and Rep-PAN into its network to enhance efficiency. Chowdhury *et al*. (2024) [28] used the YOLOv6 to detect oil palm fruit ripeness levels. Having reported that YOLOv7, with its deeper Extended-ELAN structure, outperformed YOLOv4 and YOLOv5 by Wang *et al*. (2023) [29], Zhang *et al*. (2022) [17] used the algorithm to detect dragon fruit, and Chen *et al*. (2022) [30] modified it with the added Convolutional Block Attention Module (CBAM) for citrus detection. PSP-Ellipse method was added to YOLOv7 by Zhou *et al*. (2023) [31] to further detect the endpoints of the dragon fruit after its localization and classification, and Tang *et al*. (2023b) [32] reported YOLOv7-plum to detect plum fruits quickly and accurately in a complex orchard environment. By using EfficientNet-B0 with CBAM, Tang *et al*. (2024) [33] expanded on YOLOv7 and proposed YOLOC-tiny to detect citrus fruit at different maturity levels. Likewise, Chen *et al*. (2024) [34] introduce MTD-YOLO to achieve three tasks of cherry tomato detection and fruit and bunch ripeness grading.

The introduced YOLOv8 (Jocher *et al*., 2023) [35] is an anchor-free detector that leverages on C2f modules and a decoupled head for improved detection performance. For this reason, Xiao *et al*. (2024) [36] applied the YOLOv8 algorithm for fruit ripeness identification, while Ang *et al*. (2024) [37] revealed the modified version called YCCB-YOLO to detect young citrus fruits on trees. Additionally, Li *et al*. (2023) [38] leveraged the MHSA mechanism to enhance the backbone of the YOLOv8 for tomato maturity grading and counting; and Yang *et al*. (2023) [39] incorporated a Swin-Transformer with the YOLOv8 to increase strawberry detection accuracy. The latest YOLOv9 by Wang *et al*. (2025) [40] is built upon YOLOv7. It leverages the General ELAN framework and programmable gradient information (PGI) to enhance both the efficiency and accuracy of object detection. Ye *et al*. (2024) [41] proposed CR-YOLOv9 based on modified YOLOv9 to detect strawberry fruit maturity with high accuracy and rapid speed. Li *et al*. (2024) [42] developed lightweight D3-YOLOv10 based on modified YOLOv10 (Wang *et al*., 2024) [43] to identify tomatoes in facility situations. Similarly, Fu *et al*. (2024) [44] added a squeeze-and-excitation (SE) attention mechanism into the YOLOv10 network to create MSOAR-YOLOv10 for multi-scale occluded apple detection and improved harvest robotics. Sapkota and Karkee (2024) [45] compared the detection performance of the recently released YOLOv11 (Khanam and Hussain, 2024) [46] to YOLOv8 for both occluded and non-occluded immature green fruits in a complex orchard setting, while Sapkota *et al*. (2024) [47] employed LLM-generated datasets for YOLOv11 and YOLOv10 to enhance apple detection using machine vision sensors. As expected, the mentioned fruit detectors developed via different YOLO variants, including the mainstream, were able to achieve excellent variation in detection performance.

Nevertheless, the factors that affect fruit detection when dealing with different types of fruit collections, mainly fruit characteristics and environmental conditions still remain a significant challenge. Fruit characteristics like color and texture vary between fruits. Some fruits have similar colors to their surroundings, and size and shape too might be round, elongated, and so on, fruits in dense status, and some fruits grow in clusters, making them difficult to detect. Environmental conditions such as illumination, occlusion by leaves, branches, or other fruits, and weather conditions complicate fruit detection. Fruit detection performance issues such low speed, low accuracy, and high computing cost continue to be major obstacles. Furthermore, there are limited references on fruit detection in the newly published YOLO-mainstream variants, particularly using the anchor-based method to attest performance. And most developed fruit detectors are seldom subjected to count analysis. Therefore, the main contributions of this article are as follows:

(1) Capture various collections of fruit images from their natural surroundings to build a CFruit image dataset, paying specific attention to characteristics that impede fruit detection to ensure robustness.
(2) The integration of newly designed C2fR, MPC, and SPPR modules into the Backbone network, along with the incorporation of C2fR into the Neck network of YOLOv5, aims to develop an efficient and lightweight YOLOcF fruit detector.
(3) The validation of the created CFruit image dataset using the YOLOcF fruit detector for target detection and count analysis.
(4) The comparison of target detection performance between the YOLOcF and other YOLO-mainstream variants for the most efficacious architecture for application.

## Methodology

### CFruit image dataset

**Fig 1:**
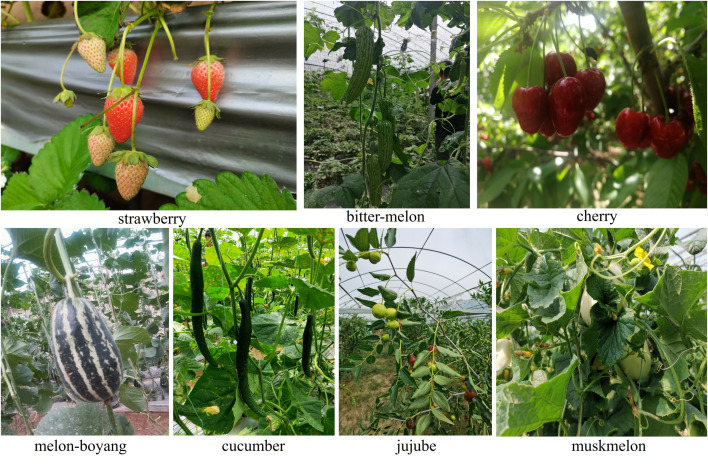
The image samples of strawberry, bitter-melon, cherry, melon-boyang, cucumber, jujube, and muskmelon fruit contained in the CFruit image dataset.

The images of fruit used for this paper were captured using a regular digital camera with a 3968 × 2976 pixels resolution in the morning, noon, and afternoon from different greenhouse locations in Houcheng town, Jinzhong, Shanxi, China. These greenhouses are open to the public without requiring permits to collect images of fruit. With special attention to environmental factors such as high-light, low-light cloudy, reflections, and shadows, including fruit complex conditions: fruit clusters, leaf occlusion, overlap, backlight, dense targets, branch occlusion, earth background, similar background, sky background, fruit size variation, front light, side light, and others, to foster the fruit detector’s robustness, seven classes of fruit were taken. The classes of images as shown in Fig 1 are strawberry, bitter-melon, cherry, melon-boyang, cucumber, jujube, and muskmelon. The constructed CFruit image dataset, having a total of 4950 images, contained JPG image format, randomly divided 80% train set and 20% valid set, and YOLO annotated format files. In addition, an mp4 video of each fruit class was recorded, and frames were extracted for unbiased evaluation, taken as a test set. Table 1 provides the dataset details.

**Table 1:** Details of CFruit image dataset.

CFruit	Train	Valid	Images	Test
Bitter-melon	532	133	665	1763
Cucumber	532	132	664	2103
Cherry	374	100	474	1567
Jujube	821	205	1026	1437
Melon-boyang	588	148	736	2167
Muskmelon	324	080	404	1346
Strawberry	785	196	981	1015
**Total**	**3956**	**994**	**4950**	**11398**

### YOLOv5

**Fig 2:**
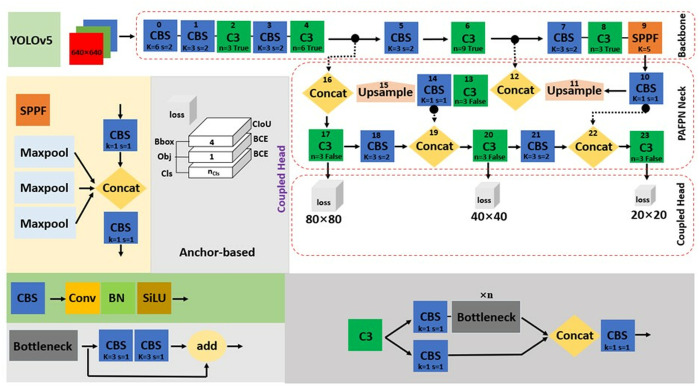
Detail of anchor-based YOLOv5.


The original YOLOv5 subjected to improvement is depicted in Fig 2. It is divided into the input, Backbone, Neck, and Coupled Head networks. The input includes adaptive anchor, mosaic data augmentation, and adaptive image scaling (0.33 depth and 0.25 width) for YOLOv5n. The Backbone consists of convolution‒batch normalization‒SiLU (CBS), C3 and SPPF modules used to accumulate fine-grained images and for feature map extraction. The PAFPN as Neck combines the extracted feature maps from the Backbone for multiscale feature fusion, before sending the integrated feature maps to the Coupled Head. The Coupled Head derives its predictions from the anchor boxes for target detection and generates the class, score, and location of targets. It also applied a complete intersection-over-union (*CIoU*) loss function (Zheng *et al*., 2019b) [48] for bounding box (Bb) and binary cross-entropy (*BCE*) loss for classification (Cls) and objectness (Obj) as described in Fig 2. *CIoU* loss enables speed convergence and accuracy localization, define by Eq. 1 with attention to overlap area (*S*), centroid distance (*D*) and aspect ratio (*V*) of the predicted box (*B*) and real box (*B*^*gt*^). *BCE* loss (Lawal *et al*., 2023a) [21] can be defined as Eq. 2, where y is the label for output range (0–1) through sigmoid, and *p*(*y*) is the predicted probability for all *N* points.

$$L_{CIoU}=S\left(B,B^{gt}\right)+D\left(B,B^{gt}\right)+V\left(B,B^{gt}\right)$$ (1)

$$BCE=-\frac{1}{N}\sum_{i-0}^{N}y_{i}*\log(p(y_{i}))+(1-y_{i})*\log(1-p(y_{i}))$$ (2)

### YOLOcF

**Fig 3:**
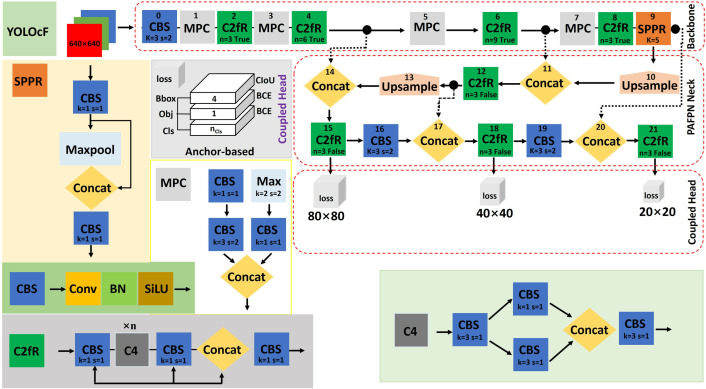
Detail of anchor-based YOLOcF.

The proposed YOLOcF (cF: collection Fruit), as shown in Fig 3, improves on the YOLOv5 framework with inspiration drawn from improved YOLOv5s (Lawal *et al*., 2023b) [24], YOLO series (Lawal, 2024) [26], and YOLOv8 (Jocher *et al*., 2023) [35]. The YOLOv5 framework was chosen for improvement because of its advantages of faster training, a lower memory footprint, the ability to detect dense objects in complex scenes, and certain application scenarios, such as on mobile devices or systems with limited resources. Similar to YOLOv5n, YOLOcF fruit detector consists of the input, Backbone, Neck, and Coupled Head networks. Although the input information for adaptive anchor, mosaic data augmentation, and adaptive image scaling (0.33 depth and 0.25 width) remains unchanged, the Backbone comprises CBS, MPC, C2fR, and SPPR modules for the extraction of feature maps. The CBS shown in Fig 3 is used for downsampling feature maps. It is a convolution followed by batch normalization activated with SiLU. Similarly, the MPC is also used for feature map downsampling, but takes the feature concatenation of two CBS, whose information comes from a CBS, and maxpooling (Maxpool). The introduced feature concatenation enables information sharing between complementary features of the low and high layers, according to Lawal *et al*. (2023b) [24]. MPC reduces the number of parameters and computation costs and increases accuracy. The idea of C2fR was created from C2f in YOLOv8, where the C4 module was added to the original network. The C4 replaces the Bottleneck in C2f of YOLOv8. The C4 module for feature map extraction embedded the feature concatenation of two CBS taken from a split feature of CBS before information was passed to the last CBS, as shown in Fig 3. The C2fR, which consists of C4 and three CBS, enables more learning of features towards an increase in accuracy. The complementary features of two CBS with C4 in the middle are concatenated before information is shared with the final CBS. The introduced SPPR was designed to replace the SPPF module used by YOLOv5 and YOLOv8. Its incorporation into the Backbone of YOLOcF was to speed up the computation and reduce the loss of features during learning while maintaining enhancement of feature expression ability. SPPR consists of a single maxpooling concatenated with a CBS followed by a CBS. As shown in Fig 3, PAFPN as Neck was adopted, whose arrangement is similar to that of YOLOv8, except for its C2f module, which is replaced by the C2fR. This is to promote excellent feature extraction while curbing gradient information duplication and reducing the number of parameters. Just like YOLOv5, YOLOcF used a Coupled Head, whose losses are defined in Eq. 1 and 2. At different scales through the Neck, the Couple Head of YOLOcF can detect large, medium, and small targets within an image.

### Experiment details

The experiments of YOLOcF, including other YOLO-mainstream variants, were validated on CFruit image dataset using the anchor-based YOLOv5 platform, with hardware and environment details in Table 2. Random initialization training from scratch was applied as all the YOLO variants received an input image of 640 × 640 × 3 pixels, 16 batches, 200 epochs, 0.937 momentum, 0.015 hue, 0.7 saturation, 0.4 value, 0.1 translate, 0.5 scale, 1.0 mosaic, and other default parameters. Initially, a study was conducted on the YOLOcF fruit detector, as depicted in Table 3, to validate the most effective contributing modules to its performance. With reference to Fig 3, the YOLOcF1, YOLOcF4, and YOLOcF5 corresponding to C2f, C3, and C2fR replace the 2^nd^, 4^th^, 6^th^, and 8^th^ layers. For YOLOcF2, C3 was placed in the 2^nd^ and 6^th^ layers, and C2f in the 4^th^ and 8^th^ layers. In YOLOcF3, C3 was placed in the 2^nd^ and 6^th^ layers, and C2fR in the 4^th^ and 8^th^ layers.

**Table 2:** The hardware and environment details of experiment.

Hardware	Configure	Environment	Version
System	Ubuntu20.04	Python	3.8.16
CPU	Core i7-12700F	Conda	23.1.0
GPU	RTX5000 (16G)	PyTorch	1.12.1
RAM	32G	CUDA	11.3.1
Hard-disk	2.0T	CUDNN	8.8.0

**Table 3:** Study on YOLOcF using different modules.

YOLOcF	C3	C2f	C2fR	PAFPN	Loss
YOLOcF1	×	✓	×	C2fR	CIoU
YOLOcF2	✓	✓	×	C2fR	CIoU
YOLOcF3	✓	×	✓	C2fR	CIoU
YOLOcF4	✓	×	×	C2fR	CIoU
YOLOcF5	×	×	✓	C2fR	CIoU

### Evaluation metrics

The evaluation metrics of the YOLOcF fruit detector compared to other YOLO-mainstream variants are mainly based on precision (*P*), recall (*R*), and mean average precision (*mAP*), where *TP*, *FP* and *FN* are the number of true positives, false positives and false negatives, respectively. *C* is the total number of classes in the dataset. *Params* defines the detector network complexity and *GFLOPs* is the number of floating-point arithmetic operations, where *i* is the input-size, *k* is the convolution kernel-size, *o* is the output-size and *H* × *W* is the size of the outputted feature map. The speed is in frames per second (*fps*), where *T*_*pre*_ is the image preprocessing time (ms), *T*_*infer*_ is the algorithm inference time (ms), and *T*_*post*_ is the post-processing time (ms).


$$P=\frac{TP}{TP+FP}$$ (3)

$$R=\frac{TP}{TP+FN}$$ (4)

$$mAP=\frac{\sum {\mathrm{\backslash nolimits}}_{1}^{C}\int_{0}^{1}P(R)dR}{C}$$ (5)

params=[i×(k×k)×o]+o (6)

GFLOPs=H×W×params (7)

$$fps=\frac{1000}{T_{pre}+T_{\inf er}+T_{post}}$$ (8)

The counting performance of all the YOLO variants on the CFruit image dataset is crucial in assessing their accuracy and reliability. The three-evaluation metrics, including Mean Absolute Error (*MAE*), Root Mean Squared Error (*RMSE*), and R-squared (*R*^*2*^) score, were explored for this purpose, where *ŷ* is the predicted value of *y* and *ȳ* is the mean value of *y*. These metrics evaluate the agreement between the predicted and ground-truth values. The *MAE*, which is less sensitive to outliers, provides a clear measure of the average prediction error; the *RMSE* measures the standard deviation of residuals in the dataset; and *R*^*2*^ quantifies the goodness of fit between the dependent variable that is predictable from the independent variables. Here, the lower values of *MAE* and *RMSE* implies higher accuracy of a YOLO variants detector. However, a higher value of *R*^*2*^ is considered desirable.


$$MAE=\frac{1}{n}\sum_{i=1}^{n}\left|{\hat{y}}_{i}-y_{i}\right|$$ (9)

$$RMSE=\sqrt{\frac{1}{n}\sum_{i=1}^{n}{\left(y_{i}-{\hat{y}}_{i}\right)}^{2}}$$ (10)

$$R^{2}=1-\frac{{\sum {\mathrm{\backslash nolimits}}_{i=1}^{n}\left(y_{i}-{\hat{y}}_{i}\right)}^{2}}{\sum {\mathrm{\backslash nolimits}}_{i=1}^{n}{\left(y_{i}-{\bar{y}}_{i}\right)}^{2}}$$ (11)

## Results and discussion

### Study on YOLOcF

**Fig 4:**
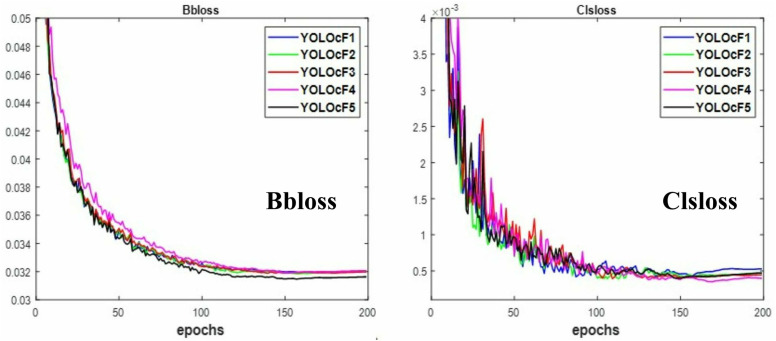
The obtained loss of YOLOcF for Bbloss and Clsloss under study.

The study carried out enables the best-performing experiments considered for the YOLOcF fruit detector. The validation loss presented in Fig 4 predicts the performance of experiments while training. The Bbloss measures the actual position of the target fruit in an image, while the Clsloss measures the associated class of the target fruit. Both validation losses exhibit a similar, closely decreasing pattern. However, the level of loss in Bb is less than in Cls, indicating the complexity of the fruit’s natural environment. Meanwhile, the level of deeper neural network noticed in YOLOcF5 is lower than YOLOcF1 to YOLOcF4, as depicted in Fig 4. The decreasing validation loss of algorithm learning resulted in an increasing *mAP*. For this reason, the *mAP* achieved by YOLOcF5, as illustrated in Table 4, is more accurate than that of YOLOcF1 to YOLOcF3, although comparable to YOLOcF4, exhibiting dependable *P* and *R* detection metrics. The incorporated C2fR module in YOLOcF5 facilitated the learning of multi-scale features and extracting contextual information from images to improve accuracy. Moreover, the computation cost of YOLOcF5 in terms of *params*, size, and *GFLOPs* is slightly higher than YOLOcF1 to YOLOcF4, having traded off for its *mAP*. Nevertheless, the tested *fps* of YOLOcF5 is faster than YOLOcF1, YOLOcF2, and YOLOcF4, but not for YOLOcF3. Because YOLOcF3 was noted to have the least computation cost compared to other experiments, as indicated in Table 4. The overall detection performance of YOLOcF5 after this study is outstanding compared to YOLOcF1 to YOLOcF4, and it is best considered for YOLOcF fruit detector. Furthermore, the close proximity in detection performance of YOLOcF1 to YOLOcF5 indicates that the C2fR module integrated into their Neck networks is effective for accuracy. Their speed can be attributed to the added MPC module of Fig 3 for downsampling. This study provides valuable insights for the future improvement and optimization of YOLOcF.

**Table 4:** The detection performance of YOLOcF under study.

YOLOcF	*Params* (×10^6^)	*Size* (×10^6^)	*GFLOPs*	*P*%	*R*%	*mAP*%	*fps*
YOLOcF1	2.1	4.5	5.1	91.7	79.5	89.0	204
YOLOcF2	2.1	4.5	5.0	90.6	80.3	88.7	222
YOLOcF3	2.0	4.4	5.0	92.6	78.3	88.9	345
YOLOcF4	2.0	4.4	4.9	91.6	80.6	89.4	213
YOLOcF5	2.1	4.6	5.2	90.1	80.8	89.4	323

### Detection performance

**Fig 5:**
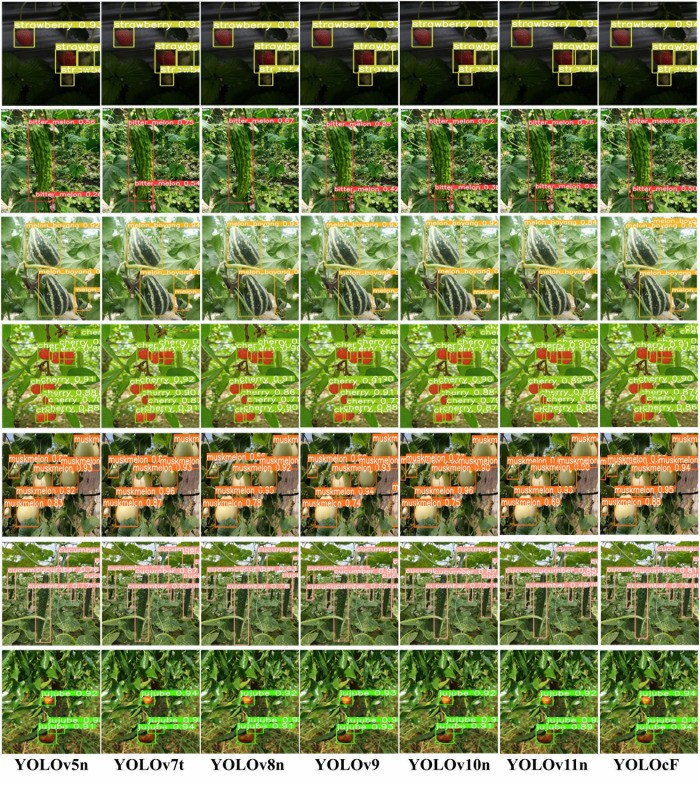
The fruit detected on YOLOv5n, YOLOv7t, YOLOv8n, YOLOv9, YOLOv10n, YOLOv11n, and YOLOcF using the test set in the CFruit dataset.

According to Fig 5 for the evaluated test set in the CFruit dataset, a number of targets were detected using YOLOv5n (Jocher *et al*., 2022) [15], YOLOv7t (Wang *et al*., 2023) [29], YOLOv8n (Jocher *et al*., 2023) [35], YOLOv9 (Wang *et al*., 2025) [40], YOLOv10n (Wang *et al*., 2024) [43], YOLOv11n (Khanam and Hussain, 2024) [46], and the YOLOcF fruit detector. Nevertheless, the detected fruit targets were distinguished with varying confidence scores, missed detection, and incorrect detection. Some portions of Fig 5 for YOLOv5n, YOLOv8n, YOLOv10n, and YOLOv11n indicate both incorrect and missed detection compared to YOLOv7t, YOLOv9, and YOLOcF. This suggests that architectural limitations, such as the difficulty of detecting very small targets, overlapping targets, or targets with unusual aspect ratios, were responsible for missed and incorrect detection. Meanwhile, it was observed that confidence scores of YOLOcF and YOLOv9 are higher than those of YOLOv5n, YOLOv7t, YOLOv8n, YOLOv10n, and YOLOv11n. Table 5 provides a better description since it is difficult to estimate the detection performance using the detected fruit targets in the images.

**Table 5:** Detection performance of YOLOcF compared to other YOLO-mainstream variants.

Variants	Computation cost			Accuracy			Speed			
	*Params* (×10^6^)	*Size* (×10^6^)	*GFLOPs*	*P%*	*R%*	*mAP%*	*T*_*pre*_	*T*_*infer*_	*T*_*post*_	*fps*
YOLOv5n	2.2	4.8	5.3	91.1	79.2	88.6	0.2	2.6	1.3	244
YOLOv7t	6.0	12.4	13.1	90.8	79.6	88.3	0.2	3.6	1.4	196
YOLOv8n	2.3	4.8	5.2	89.6	79.5	88.1	0.2	2.6	1.7	222
YOLOv9	2.6	52.5	92.3	91.5	81.3	90.8	0.2	16.5	1.2	56
YOLOv10n	1.8	4.1	4.9	91.3	79.1	88.7	0.2	2.7	1.5	227
YOLOv11n	2.0	4.4	4.1	90.2	79.8	88.6	0.2	1.9	1.4	285
YOLOcF	2.1	4.6	5.2	90.1	80.8	89.4	0.2	2.1	0.8	323

First, YOLOcF is less than YOLOv5n, YOLOv7t, YOLOv8n, and YOLOv9, but more than YOLOv10n and YOLOv11n, according to the computation cost that was collected and displayed in Table 5. In comparison to YOLOv5n, YOLOv7t, YOLOv8n, and YOLOv9, the percentage reduction value of YOLOcF is 4.5%, 65%, 8.7%, and 19.2% smaller via *params* and 1.9%, 60.3%, 0%, and 94.4% smaller via *GFLOPs*. Because of this, YOLOcF is lighter than other YOLO variants, which makes it easier to deploy mobile or low-power computer devices and allows for faster training. Unfortunately, the computation cost of YOLOv10n and YOLOv11n is less than YOLOcF, where 14.3% and 4.8% are via *params*, and 5.8% and 21.2% are via *GFLOPs*, respectively. Second, YOLOcF is 0.8%, 1.1%, 1.3%, −1.4%, 0.7%, and 0.8% more accurate than YOLOv5n, YOLOv7t, YOLOv8n, YOLOv9, YOLOv10n, and YOLOv11n, respectively, utilizing the *mAP* because of its *P* − *R* global relationship. This is to say that YOLOcF demonstrated a higher detection superiority than other YOLO variants except for YOLOv9. The higher detection of YOLOv9 can be attributed to its high computation cost through its complex network, having adaptive image scaling of one for both width and depth. This adaptive scaling ensures sufficient resolution to detect fine details and better localize targets. The lowest *mAP* of YOLOv8n in Table 5 when compared to other YOLO variants explained its missed detection and lower confidence score in Fig 5. Third, the percentage increase in *fps* for speed performances, as shown in Table 5, indicates that YOLOcF is 32.4%, 64.8%, 45.5%, 476.8%, 42.3%, and 13.3% faster than YOLOv5n, YOLOv7t, YOLOv8n, YOLOv9, YOLOv10n, and YOLOv11n, respectively. Hence, the speed of YOLOcF surpasses that of other YOLO variants, supporting its lightweight design and allowing for real-time fruit detection without compromising accuracy.

The robustness performance of YOLOcF relative to other YOLO variants on the validation set of the CFruit image dataset was substantiated using count analysis, as presented in Table 6, utilizing Eq. (9)-(11). The measured *MAE* indicates that YOLOv10n has a lower value than YOLOv7t, followed by YOLOv8n, YOLOv11n, YOLOcF, YOLOv5n, and YOLOv9. Conversely, the *RMSE* value for YOLOcF is smaller than that of YOLOv7t, YOLOv8n, YOLOv11n, YOLOv10n, YOLOv5n, and YOLOv9. The lower values of *MAE* and *RMSE* constituted higher accuracy, but their trends among the YOLO variants are inconsistent. The application of *R*^*2*^ is considerable because it provides goodness of fit between variables. YOLOcF has the greatest *R*^*2*^ of 0.442, exceeding YOLOv7t at 0.427, YOLOv11n at 0.411, YOLOv9 at 0.394, YOLOv8n at 0.339, YOLOv10n at 0.373, and YOLOv5n at 0.372. Therefore, YOLOcF fruit detector is robust against fruit complex environments and reliable compared to other YOLO- mainstream variants.

**Table 6:** Count analysis tested on YOLO variants.

Variants	*MAE*	*RMSE*	*R* ^*2*^
YOLOv5n	2.10	10.60	0.372
YOLOv7t	1.95	9.31	0.427
YOLOv8n	1.96	9.66	0.399
YOLOv9	2.15	10.91	0.394
YOLOv10n	1.93	10.33	0.373
YOLOv11n	1.97	10.13	0.411
YOLOcF	1.99	9.28	0.442

## Conclusions

With special attention to fruit complex natural conditions, a collection of fruit images called the CFruit image dataset was created and validated on the newly developed YOLOcF fruit detector. The YOLOcF detector, an improved anchor-based version of YOLOv5, consists of CBS, MPC, C2fR, and SPPR modules for the extraction of feature maps in the Backbone network, as well as CBS and C2fR modules for multiscale feature fusion in the Neck network. The YOLOcF was evaluated using computation cost, *mAP*, speed, and count analysis and compared to the YOLOv5n, YOLOv7t, YOLOv8n, YOLOv9, YOLOv10n, and YOLOv11n of YOLO variants. The obtained computation cost of YOLOcF is lower than other YOLO variants, but not for YOLOv10n and YOLOv11n. Meanwhile, the *mAP* of YOLOcF exceed YOLO-mainstream variants, but not for YOLOv9. The YOLOv9 network’s high *mAP* is a result of its adaptive image scaling of one for both width and depth. Nevertheless, the speed of YOLOcF surpasses that of other YOLO variants. YOLOcF fruit detector is robust against a fruit complex environment compared to other YOLO variants for having the highest *R*^*2*^ value from count analysis. Therefore, YOLOcF holds significant potential for generalized fruit detection due to its ability to balance speed and accuracy. It’s a lightweight detector for easy mobile device deployment and faster training. Future research would need to improve the performance of YOLOcF and validate it with other diverse image datasets from multiple classes. And also, to investigate the anchor-free method’s ability to handle dense fruit detection scenarios in comparison to anchor-based detectors.
